# The Spinal Dural Arteriovenous Fistula in a Patient With Metastatic Renal Cell Carcinoma

**DOI:** 10.7759/cureus.15303

**Published:** 2021-05-28

**Authors:** Dan Tong Jia, Caitlin S Jacobs, Mengxuan Tang, Ali Shaibani, Rimas V Lukas

**Affiliations:** 1 Neurology, Northwestern University, Feinberg School of Medicine, Chicago, USA; 2 Neuroradiology, Northwestern University, Feinberg School of Medicine, Chicago, USA; 3 Neuro-Oncology, Northwestern University, Feinberg School of Medicine, Chicago, USA

**Keywords:** spinal dural arteriovenous fistula, congestive myelopathy, embolization, renal cell carcinoma, vascular endothelial growth factor, epidural metastasis

## Abstract

Spinal dural arteriovenous fistula (SDAVF) is an elusive and underdiagnosed disease. Congestive myelopathy occurs from increased venous pressure transmitted by the fistula between a radiculomeningeal artery and the spinal venous plexus. While its cause remains unknown, associations between SDAVF and hyper-vascular states have been reported. We present the first documented case report of a de novo SDAVF diagnosis in a patient with active renal cell carcinoma (RCC) metastasis to the spinal epidural space and review the literature.

## Introduction

Spinal dural arteriovenous fistulas (SDAVFs) are the most common spinal vascular malformation, however, SDAVFs are rare with an incidence rate of five to 10 cases per million in the general population and are likely underdiagnosed [1,2]. A recent meta-analysis of SDAVF patients identified several epidemiologic risk factors, including middle age (mean age of diagnosis is 55-60 years old) and male sex (ratio 5:1), but little is known about its pathogenesis or additional disease associations [3]. Interestingly, benign meningeal tumors have been associated with intracranial dural arteriovenous fistulas thought secondary to their local angiogenesis [4,5]. We present the first reported case of spinal dural arteriovenous fistula diagnosed in the presence of the renal cell carcinoma metastases to the vertebral bone and spinal dura. 

## Case presentation

A 69-year-old man presented with progressive lower extremity weakness over the past six months, as well as a new focal left-arm weakness in the past month. His medical history was significant for metastatic clear cell renal carcinoma (RCC), resected locally, and recurred three years prior with metastasis to the liver, lungs, and thoracic vertebrae. Progression of disease occurred on pazopanib, a multi-kinase inhibitor, so the patient was switched to nivolumab, a PD-1 antibody. Throughout the treatment, he was ambulating four miles daily until he developed insidiously progressive bilateral leg weakness over six months, worsened by prolonged upright standing and exacerbated by increasing falls where his legs give out on him. As such, he remained chair-bound for the week prior to the presentation. No reports of numbness, paresthesias, or pain. Lumbar MRI obtained five months prior to presentation was normal. He subsequently developed a new left-hand weakness which prompted him to present to the hospital. Examination revealed decreased (three of five) left elbow extension, wrist extension, finger extension, and intrinsic hand muscles. The bilateral hip flexor, abductor and adductor, and knee flexor weakness (four of five) were demonstrated with no overt fatigability. He displayed normal muscle tone and diminished upper and lower extremity reflexes bilaterally. Vibration sense was absent up to the knees without the involvement of his upper extremities. No cognitive impairment, bulbar weakness, nystagmus, or dysmetria were elicited; the patient denied any incontinence or saddle anesthesia. The serologic evaluation demonstrated no evidence of myopathic, metabolic, systemic infectious, or inflammatory disorders.

Cerebrospinal fluid (CSF) analysis demonstrated an opening pressure of 13 cm H_2_O, zero white blood cells, normal glucose, elevated protein (74 mg/dL, normal is 15-45 mg/dL), and no malignant cells on cytology. CSF paraneoplastic panel and serum neuromyelitis optica antibodies were negative.

**Figure 1:**
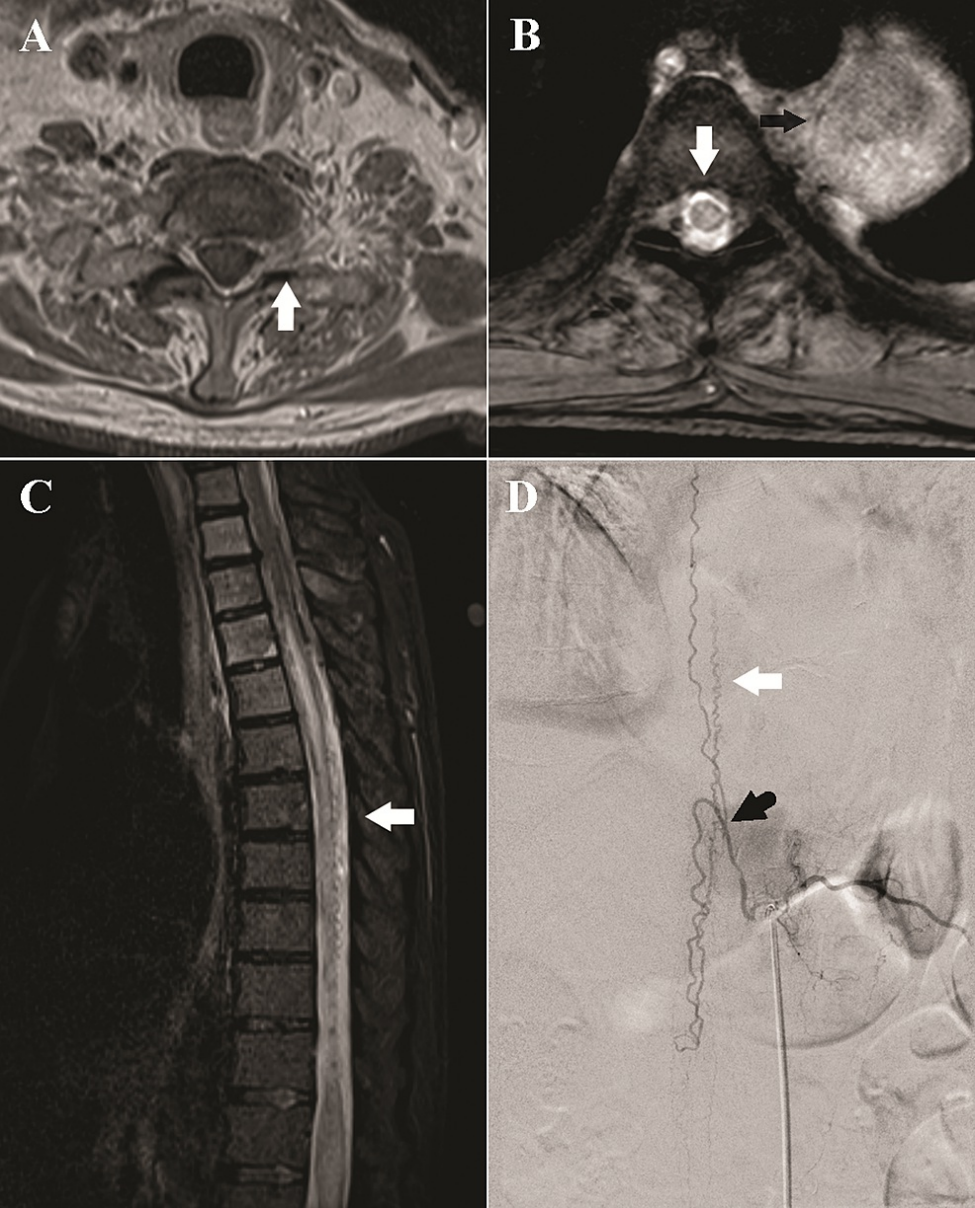
Thoracic Spine Imaging (A) Axial T1-weighted Gd+ images of the cervical spine at C8-T1 demonstrate the circumferential epidural enhancement and widening of the left neuroforamen (white arrow), concerning the metastatic disease. (B) Axial multiple echo data image combination (MEDIC) image at T5-6 demonstrates the central cord signal (white arrow). A large mass is seen along with the left aspect of the vertebral column (black arrow). ​(C) Sagittal short T1 inversion recovery (STIR) sequence of the thoracic spine demonstrates longitudinally extensive cord signal from T4 to the conus medullaris, consistent with venous congestion. There are prominent flow voids along the posterior aspect of the cord in the lower thoracic spinal canal (white arrow). (D) Angiography of the left T11 segment intercostal artery demonstrates evidence of a dural arteriovenous fistula with engorgement of the radicular vein (black arrow), which subsequently drains into a dilated spinal vein cranially and caudally (white arrow), corresponding to the flow voids visualized on MRI.

Repeated MRI of the brain and spinal cord demonstrated a metastatic lesion in the upper thoracic vertebrae extending into the left spinal foramina at the lower cervical levels compressing the C7-T1 ventral nerve roots and electromyography confirmed hyper-acute denervation of the left-sided C7-T1 innervated muscles (Figure 1A). Additionally, a prominent T2 hyperintense signal within the cord was visualized extending from the T4 level to the conus medullaris with associated cord expansion (Figure 1B). This raised concerns for venous myelopathy, which was not previously visualized on the MRI lumbar spine obtained five months prior to presentation. Concentrically enhancing epidural metastases were also visualized at T6 but they did not cause cord compression. Peri-medullary flow voids and early venous filling on time-resolved sequences further suggested SDAVF as the etiology of the progressive venous myelopathy and the patient’s worsening bilateral lower extremity weakness (Figure 1C).

Spinal angiography identified a type I spinal dural arteriovenous fistula supplied by branches of the left T11 intercostal artery (Figure 1D). The left T11 intercostal artery was successfully catheterized, and the DAVF embolized with n-butyl cyanoacrylate glue, with penetration of the proximal draining vein. The patient’s postoperative course was neurologically uncomplicated and his lower extremity strength and sensory exam improved to near baseline by one month following hospitalization.

## Discussion

Our patient with metastatic clear cell RCC presented with multiple spinal pathologies. The first was bone metastasis resulting in compressive C7-T1 motor polyneuropathy. The second was SDAVF causing progressive thoracolumbar venous myelopathy. Interestingly, the third was epidural metastases with concentric enhancement found at the T6 level. Although epidural metastases are a rare cause of myelopathic symptoms in cancer, imaging without appreciable thecal sac narrowing, negative cytology, and normal opening pressure on lumbar puncture suggest they are not the primary culprit for his gait difficulties [6]. Additional vigilance is necessary to rule out additional paraneoplastic and autoimmune etiologies of myelopathy, polyneuropathy, or neuromuscular junction diseases in a cancer patient that is undergoing active immunotherapy. In our clinical case, the non-inflamed CSF fluid and neurophysiologic evaluation did not demonstrate a polyneuropathic process in the lower extremities.

Spinal dural arteriovenous fistulas (SDAVFs) are the most common spinal vascular malformation, accounting for 50-85% of all vascular spinal malformations. The reported incidence of SDAVF is five to 10 cases per million in the general population with men affected four to five times as frequently as women. The vast majority of lesions occur between T6 and L2 and initial symptoms are often nonspecific and include gait difficulty (51-80%), symmetric or asymmetric motor/sensory deficits, radicular pain, bowel/bladder difficulty (up to 75%), and sexual dysfunction (17%) [2]. Most patients progress indolently for one to three years before diagnosis, and the disease likely remains under-diagnosed because of its insidious course and non-specific symptoms that mimic degenerative spinal stenosis, polyradiculopathies, or anterior horn cell disorders [3].

Spinal MRI can demonstrate homogenous T2 hyperintensity in the central spinal cord spanning an average of five to seven vertebral levels, commonly with conus involvement and expansion, which is suggestive of congestive myelopathy. These findings can also be seen in immune-mediated and neoplastic etiologies. T2 hypointense “flow-voids” on the dorsal surface of the spinal cord can be seen in 35-91% of patients and represent tortuously and dilated peri-medullary veins [3]. CT angiogram and MR angiogram can demonstrate early venous filling and better localize the level of the shunt [2]. CSF analysis will often demonstrate elevated protein but is neither sensitive nor specific for SDAVF; rather, its role is crucial for excluding alternate diagnoses. The gold diagnostic standard remains spinal angiography, which allows for precise characterization of location and severity. Spinal venous hypertension is evidenced by delayed venous return on anterior spinal artery contrast injection, while the early venous return will be visualized on injecting the segmental artery harboring the SDAVF.

Treatment for SDAVF is a rapidly changing field. Traditional therapy involves surgical ligation of the draining vein to the fistula, while newer endovascular embolization of the SDAVF is performed using a glue solution or co-polymer to achieve permanent occlusion in 61-83% of patients [7,8]. Robust comparison between surgery and embolization is difficult in the setting of the heterogeneity in fistula anatomy and embolizing materials including polyvinyl alcohol, cyanoacrylate mixture to newer Onyx embolic agent. A large single-center retrospective study of 63 SDAVF patients over 20 years discovered that both treatments have similar outcomes, but surgical patients require less frequent re-intervention (21% vs 31%) while endovascular patients have shorter hospital stays (3.1 days vs 9.8 days) [9]. The symptomatic outcomes are quantified with the Aminoff-Logue disability scale (ALDS), where gait and micturition dysfunction are scored on a numeric scale of five and three, respectively, with zero representing the normal function. An average ALDS reduction of one to two is expected in 55% of patients, with 34% achieving symptomatic stability and 11% suffering deterioration [10]. Improvement in motor deficits is more common than sensory or incontinence symptoms, 63% vs 40% and 44%, respectively. Fistula location also correlated with symptomatic improvement. When compared to upper thoracic (70%) or lumbar (78%) localizations, 95% of SDAVF patients with lower thoracic spine fistulas (T9-T12) show improvement in at least one symptom [11]. This may reflect varied lengths of affected cord or preserved vascular perfusion in the lower thoracic region from the artery of Adamkiewicz. Conversely, patients with a large burden of pre-treatment disability are less likely to recover. Grandin et al. suggest up to 30% of patients are already wheelchair users at the time of diagnosis and do not meaningfully regain nerve function [12].

The pathogenesis behind the fistula formation in SDVAF remains unknown. Associations between RCC and SDAVF have not yet been reported, although RCC is commonly associated with extracranial arteriovenous malformations (AVM) [13]. Interestingly, Albander et al describe a patient with RCC with extracranial metastasis who developed an intracranial arteriovenous malformation that recurred despite initial resection [14]. On the third resection of the same intracranial territory, the mass did not morphologically represent an AVM but instead stained positive for RCC biomarkers [14]. The association between AVMs and RCC is attributed to their highly vascular nature. Recent studies demonstrate that RCC uniquely elevates free vascular endothelial growth factor (VEGF) levels in the plasma, likely secondary to mutations in the Von Hippel-Lindau gene. The unshackling of the hypoxia-inducible factor pathway not only plays a crucial role in RCC tumor angiogenesis but also systemic vascular hyperplasia [15,16]. Systemic VEGF activation has been shown to contribute to the growth of intracranial dural arteriovenous fistulas (DAVF), demonstrated in both animal models and human patients [17,18]. VEGF and TIE-2 endothelial cell membrane receptors to angiopoietins are found at lower levels after DAVF embolization and are hypothesized as a biomarker of treatment response [18]. A fascinating case report by Hedjoudje et al. highlights a patient with Cowden’s disease who developed multiple SDAVFs, which is aligned with the hypervascular hypothesis of SDAVF pathophysiology [19]. Further investigations are needed to elucidate the relationship between SDAVF and mutations in angiogenesis.

## Conclusions

Spinal dural arteriovenous fistulas are underdiagnosed and can result in permanent disability if the diagnosis is delayed. Although the diagnosis and treatment methods are rapidly advancing, the pathogenesis of SDAVF is not well understood. The most prevalent theory for cranial DAVF is local venous hypertension, which is extrapolated to SDAVFs. However, recent studies in intracranial DAVF have implicated VEGF and other angiogenic molecules to the growth and indicators of disease, which, if valid in SDAVF, can provide a significant clinical and diagnostic utility. To our knowledge, we present the first reported case of SDAVF diagnosed in a patient with metastatic spinal RCC and paves the path for further investigations into angiogenesis in the pathogenesis of SDAVF.
